# 1-Ethyl-*N*′-[(*E*)-4-hydroxy­benzyl­idene]-7-methyl-4-oxo-1,4-dihydro-1,8-naphthyridine-3-carbohydrazide

**DOI:** 10.1107/S1600536809009167

**Published:** 2009-03-25

**Authors:** Farah Deeba, Misbahul Ain Khan, Muhammad Zia-ur-Rehman, Nagihan Çaylak, Ertan Şahin

**Affiliations:** a Applied Chemistry Research Centre, PCSIR Laboratories Complex, Ferozpure Road, Lahore 54600, Pakistan; b Department of Chemistry, Islamia University, Bahawalpur, Pakistan; c Department of Physics, Sakarya University, Sakarya, Turkey; d Department of Chemistry, Faculty of Science, Atatürk University, 25240 Erzurum, Turkey

## Abstract

In the crystal structure of the title compound, C_19_H_18_N_4_O_3_, the fused-ring system is essentially planar  [maximum deviation is 0.031 (2) Å] while the dihedral angle between the ring system and the benzene ring is 12.64 (6)°.The carbohydrazide H atom is involved in an intra­molecular N—H⋯O hydrogen bond, forming a six-membered hydrogen-bonded ring. The mol­ecules arrange themselves into centrosymmetric dimers by means of inter­molecular O—H⋯O hydrogen bonds.

## Related literature

For the synthesis of heterocyclic compounds, see: Chen *et al.* (2001 ▶); Zia-ur-Rehman *et al.* (2006 ▶, 2009 ▶). For their biological activity, see: Ferrarini *et al.* (2000 ▶); Gavrilova & Bosnich (2004 ▶); Goswami & Mukherjee (1997 ▶); Hoock *et al.* (1999 ▶); Mintert & Sheldrick (1995 ▶); Nakatani *et al.* (2000 ▶); Nakataniz *et al.* (2001 ▶); Roma *et al.* (2000 ▶). For similar mol­ecules, see: Catalano *et al.* (2000 ▶).
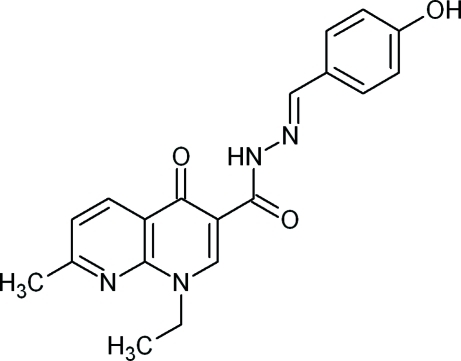


## Experimental

### 

#### Crystal data

C_19_H_18_N_4_O_3_
*M* *_r_* = 350.37
Monoclinic, 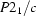

*a* = 7.6437 (2) Å
*b* = 13.3290 (2) Å
*c* = 17.2212 (4) Å
β = 98.1745 (14)°
*V* = 1736.72 (7) Å^3^
*Z* = 4
Mo *K*α radiation
μ = 0.09 mm^−1^
*T* = 293 K
0.2 × 0.14 × 0.1 mm

#### Data collection

Rigaku R-AXIS RAPID-S diffractometer
Absorption correction: multi-scan (Blessing, 1995 ▶) *T*_min_ = 0.990, *T*_max_ = 0.991
35553 measured reflections
3583 independent reflections
2597 reflections with *I* > 2σ(*I*)
*R*_int_ = 0.064

#### Refinement

*R*[*F* ^2^ > 2σ(*F* ^2^)] = 0.056
*wR*(*F* ^2^) = 0.151
*S* = 1.04
3583 reflections
244 parameters
H atoms treated by a mixture of independent and constrained refinement
Δρ_max_ = 0.15 e Å^−3^
Δρ_min_ = −0.16 e Å^−3^

### 

Data collection: *CrystalClear* (Rigaku/MSC, 2005 ▶); cell refinement: *CrystalClear*; data reduction: *CrystalClear*; program(s) used to solve structure: *SHELXS97* (Sheldrick, 2008 ▶); program(s) used to refine structure: *SHELXL97* (Sheldrick, 2008 ▶); molecular graphics: *ORTEP-3 for Windows* (Farrugia, 1997 ▶); software used to prepare material for publication: *WinGX* (Farrugia, 1999 ▶).

## Supplementary Material

Crystal structure: contains datablocks global, I. DOI: 10.1107/S1600536809009167/ng2560sup1.cif

Structure factors: contains datablocks I. DOI: 10.1107/S1600536809009167/ng2560Isup2.hkl

Additional supplementary materials:  crystallographic information; 3D view; checkCIF report

## Figures and Tables

**Table 1:** Hydrogen-bond geometry (Å, °)

*D*—H⋯*A*	*D*—H	H⋯*A*	*D*⋯*A*	*D*—H⋯*A*
N3—H3*N*⋯O1	0.91 (2)	1.91 (2)	2.660 (3)	139 (2)
O3—H3*O*⋯O2^i^	0.82	1.90	2.721 (3)	179

Symmetry code: (i) 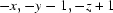.

**Fig. 1.**
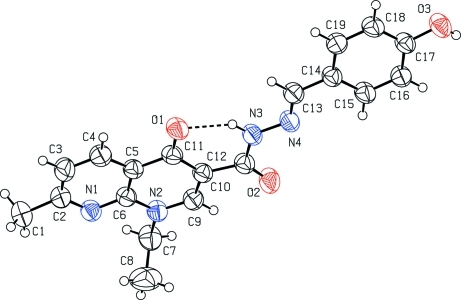
An ORTEP-3 (Farrugia, 1997) drawing of the title molecule with the atom-numbering scheme. The displacement ellipsoids are drawn at the 50% probability level.

## supplementary crystallographic information

### Comment

Derivatives of 1,8-naphthyridine have been investigated since a long ago due to their interesting complexation properties and medical uses. Such compounds are known to possess antibacterial (Chen *et al.*, 2001), anti-inflammatory (Roma *et al.*, 2000), anti-hypertensive, and anti-platelet activities (Ferrarini *et al.*, 2000). These have also been widely utilized as molecular recognition receptors for urea, carboxylic acids and guanine (Goswami *et al.*, 1997; Nakatani *et al.*, 2000). Few 1,8-naphthyridine derivatives have been reported to be excellent fluorescent markers of nucleic acids (Hoock *et al.*, 1999) and probe molecules (Nakataniz *et al.*, 2001). In addition, these have received much attention due to the possibility of their linkage with metals in several coordination modes such as monodentate, chelating bidentate, and dinuclear bridging binding fashion (Gavrilova & Bosnich, 2004; Mintert & Sheldrick, 1995).

In continuation of our work on the synthesis, biological activity and crystal structures of various heterocyclic compounds (Zia-ur-Rehman *et al.*, 2006; Zia-ur-Rehman *et al.*, 2009), we herein report the synthesis and crystal structure of the title compound (I) (Scheme and figure 1). The structure of the basic naphthyridine ring consisting of two adjoined pyridine rings is planar while carbonyl oxygen O1 on C11 is involved in intramolecular hydrogen bonding giving rise to a six-membered hydrogen bond ring (Table 1). All bond distances are essentially identical to those found in the literature (Catalano *et al.*, 2000).The molecules form centrosymmetric dimers through intermolecular O—H···O hydrogen bonds.

### Experimental

A mixture of 1-ethyl-7-methyl-4-oxo-1,4-dihydro-1,8-naphthyridine-3-carbohydrazide (10.0 mmoles; 2.46 g), 4-hydroxy benzaldehyde (11.0 mmoles; 1.34 g), *ortho* phosphoric acid (2 drops) and ethyl alcohol (20.0 ml) was refluxed for a period of two hours. After completion of the reaction as indicated by TLC, three fourth of the solvent was evaporated and the contents were cooled to room temperature. Crystals obtained were washed with cold ethanol and dried; Yield: 92%.

### Refinement

H atoms were placed in geometrically idealized positions (C—H = 0.93–0.98 Å, O—H=0.82 Å) and treated as riding, with *U*_iso_(H)=1.2*U*_eq_(C) (for methine and methylene) or 1.5*U*_eq _(methyl and hydroxyl). The N3 and C13 H atoms were located in a difference Fourier map

### Crystal data

**Table:**

C_19_H_18_N_4_O_3_	*F*(000) = 736
*M**_r_* = 350.37	*D*_x_ = 1.34 Mg m^−^^3^
Monoclinic, *P*2_1_/*c*	Mo *K*α radiation, λ = 0.71073 Å
Hall symbol: -P 2ybc	Cell parameters from 3643 reflections
*a* = 7.6437 (2) Å	θ = 2.4–26.4°
*b* = 13.3290 (2) Å	µ = 0.09 mm^−^^1^
*c* = 17.2212 (4) Å	*T* = 293 K
β = 98.1745 (14)°	Prism, light yellow
*V* = 1736.72 (7) Å^3^	0.2 × 0.14 × 0.1 mm
*Z* = 4	

### Data collection

**Table:**

Rigaku R-AXIS RAPID-S diffractometer	3583 independent reflections
graphite	2597 reflections with *I* > 2σ(*I*)
Detector resolution: 10.0000 pixels mm^-1^	*R*_int_ = 0.064
ω scans	θ_max_ = 26.7°, θ_min_ = 2.4°
Absorption correction: multi-scan (Blessing, 1995)	*h* = −8→9
*T*_min_ = 0.990, *T*_max_ = 0.991	*k* = −16→16
35553 measured reflections	*l* = −21→21

### Refinement

**Table:**

Refinement on *F*^2^	Primary atom site location: structure-invariant direct methods
Least-squares matrix: full	Secondary atom site location: difference Fourier map
*R*[*F*^2^ > 2σ(*F*^2^)] = 0.056	Hydrogen site location: inferred from neighbouring sites
*wR*(*F*^2^) = 0.151	H atoms treated by a mixture of independent and constrained refinement
*S* = 1.04	*w* = 1/[σ^2^(*F*_o_^2^) + (0.0654*P*)^2^ + 0.3979*P*] where *P* = (*F*_o_^2^ + 2*F*_c_^2^)/3
3583 reflections	(Δ/σ)_max_ < 0.001
244 parameters	Δρ_max_ = 0.15 e Å^−^^3^
0 restraints	Δρ_min_ = −0.16 e Å^−^^3^

### Special details

**Table:**

Geometry. All s.u.'s (except the s.u. in the dihedral angle between two l.s. planes) are estimated using the full covariance matrix. The cell s.u.'s are taken into account individually in the estimation of s.u.'s in distances, angles and torsion angles; correlations between s.u.'s in cell parameters are only used when they are defined by crystal symmetry. An approximate (isotropic) treatment of cell s.u.'s is used for estimating s.u.'s involving l.s. planes.
Refinement. Refinement of *F*^2^ against ALL reflections. The weighted *R*-factor *wR* and goodness of fit *S* are based on *F*^2^, conventional *R*-factors *R* are based on *F*, with *F* set to zero for negative *F*^2^. The threshold expression of *F*^2^ > 2σ(*F*^2^) is used only for calculating *R*-factors(gt) *etc*. and is not relevant to the choice of reflections for refinement. *R*-factors based on *F*^2^ are statistically about twice as large as those based on *F*, and *R*- factors based on ALL data will be even larger.

### Fractional atomic coordinates and isotropic or equivalent isotropic displacement parameters (Å^2^)

**Table:**

	*x*	*y*	*z*	*U*_iso_*/*U*_eq_	
O1	0.2080 (2)	0.01852 (11)	0.57270 (8)	0.0654 (4)	
O3	−0.1795 (2)	−0.65000 (11)	0.71180 (9)	0.0686 (4)	
H3O	−0.1729	−0.6919	0.6775	0.103*	
N1	0.4591 (2)	0.22498 (12)	0.39012 (10)	0.0556 (4)	
N3	0.1243 (2)	−0.16773 (13)	0.52592 (10)	0.0554 (4)	
N4	0.0658 (2)	−0.26173 (12)	0.54348 (10)	0.0554 (4)	
O2	0.1598 (2)	−0.20967 (11)	0.40138 (9)	0.0750 (5)	
N2	0.3819 (2)	0.05987 (12)	0.36073 (9)	0.0532 (4)	
C17	−0.1341 (3)	−0.55811 (15)	0.68611 (11)	0.0524 (5)	
C14	−0.0321 (3)	−0.36963 (14)	0.63803 (11)	0.0517 (5)	
C11	0.2538 (3)	0.03007 (14)	0.50649 (11)	0.0505 (5)	
C10	0.2423 (3)	−0.04590 (14)	0.44689 (11)	0.0493 (5)	
C16	−0.1130 (3)	−0.54200 (15)	0.60834 (11)	0.0567 (5)	
H16	−0.1315	−0.594	0.5721	0.068*	
C9	0.3081 (3)	−0.02682 (15)	0.37834 (12)	0.0544 (5)	
H9	0.3009	−0.0779	0.3413	0.065*	
C13	0.0289 (3)	−0.27273 (16)	0.61292 (12)	0.0561 (5)	
C15	−0.0647 (3)	−0.44868 (15)	0.58534 (11)	0.0590 (5)	
H15	−0.0533	−0.438	0.5329	0.071*	
C6	0.3892 (3)	0.13908 (14)	0.41286 (11)	0.0494 (5)	
C18	−0.1077 (3)	−0.48014 (16)	0.73924 (11)	0.0580 (5)	
H18	−0.1243	−0.4903	0.7911	0.07*	
C5	0.3272 (3)	0.12561 (14)	0.48473 (11)	0.0500 (5)	
C2	0.4702 (3)	0.30211 (15)	0.43905 (13)	0.0586 (5)	
C12	0.1715 (3)	−0.14835 (14)	0.45515 (12)	0.0539 (5)	
C19	−0.0567 (3)	−0.38686 (15)	0.71523 (11)	0.0562 (5)	
H19	−0.0385	−0.3348	0.7515	0.067*	
C1	0.5445 (3)	0.39767 (17)	0.41103 (15)	0.0736 (7)	
H1A	0.5764	0.3875	0.3597	0.11*	
H1C	0.6474	0.4168	0.4466	0.11*	
H1B	0.4573	0.4498	0.4089	0.11*	
C4	0.3414 (3)	0.20843 (16)	0.53477 (13)	0.0648 (6)	
H4	0.3023	0.2041	0.5834	0.078*	
C3	0.4127 (4)	0.29585 (17)	0.51221 (13)	0.0716 (6)	
H3	0.4229	0.3511	0.5456	0.086*	
C7	0.4608 (3)	0.06988 (18)	0.28730 (12)	0.0679 (6)	
H7A	0.4911	0.0038	0.2698	0.082*	
H7B	0.5691	0.1086	0.2979	0.082*	
C8	0.3387 (4)	0.1199 (3)	0.22344 (15)	0.1028 (10)	
H8B	0.3956	0.1254	0.1774	0.154*	
H8C	0.3093	0.1857	0.2403	0.154*	
H8A	0.2328	0.0808	0.2116	0.154*	
H13	0.045 (3)	−0.2191 (17)	0.6511 (13)	0.067 (6)*	
H3N	0.131 (3)	−0.1174 (17)	0.5616 (13)	0.070 (7)*	

### Atomic displacement parameters (Å^2^)

**Table:**

	*U*^11^	*U*^22^	*U*^33^	*U*^12^	*U*^13^	*U*^23^
O1	0.0978 (11)	0.0566 (9)	0.0456 (8)	−0.0032 (8)	0.0238 (8)	0.0018 (6)
O3	0.1010 (12)	0.0523 (8)	0.0579 (9)	−0.0040 (8)	0.0296 (9)	0.0053 (7)
N1	0.0569 (10)	0.0537 (10)	0.0566 (10)	−0.0026 (8)	0.0098 (8)	0.0042 (8)
N3	0.0732 (11)	0.0448 (9)	0.0499 (10)	−0.0026 (8)	0.0139 (8)	0.0024 (8)
N4	0.0676 (11)	0.0446 (9)	0.0551 (10)	−0.0016 (8)	0.0125 (8)	0.0040 (7)
O2	0.1160 (14)	0.0533 (9)	0.0596 (9)	−0.0130 (8)	0.0259 (9)	−0.0086 (7)
N2	0.0632 (10)	0.0529 (9)	0.0466 (9)	−0.0036 (8)	0.0186 (7)	−0.0010 (7)
C17	0.0623 (12)	0.0483 (11)	0.0491 (11)	0.0007 (9)	0.0159 (9)	0.0051 (8)
C14	0.0608 (12)	0.0479 (10)	0.0481 (11)	−0.0006 (9)	0.0131 (9)	0.0019 (8)
C11	0.0581 (12)	0.0503 (11)	0.0436 (10)	0.0056 (9)	0.0093 (8)	0.0023 (8)
C10	0.0561 (11)	0.0473 (10)	0.0452 (10)	0.0009 (8)	0.0090 (8)	0.0025 (8)
C16	0.0767 (14)	0.0502 (11)	0.0454 (11)	−0.0023 (10)	0.0165 (10)	−0.0024 (8)
C9	0.0658 (13)	0.0496 (11)	0.0492 (11)	−0.0001 (9)	0.0132 (9)	−0.0043 (9)
C13	0.0698 (14)	0.0492 (11)	0.0507 (12)	0.0003 (10)	0.0130 (10)	−0.0005 (9)
C15	0.0837 (15)	0.0531 (12)	0.0438 (11)	−0.0039 (10)	0.0210 (10)	0.0011 (9)
C6	0.0526 (11)	0.0486 (10)	0.0472 (10)	0.0016 (8)	0.0082 (8)	0.0019 (8)
C18	0.0748 (14)	0.0598 (12)	0.0422 (10)	0.0000 (10)	0.0186 (9)	0.0041 (9)
C5	0.0579 (11)	0.0487 (11)	0.0439 (10)	0.0003 (9)	0.0084 (8)	0.0012 (8)
C2	0.0617 (13)	0.0507 (12)	0.0619 (13)	−0.0030 (9)	0.0038 (10)	0.0032 (10)
C12	0.0644 (13)	0.0476 (11)	0.0505 (11)	0.0016 (9)	0.0109 (9)	−0.0002 (9)
C19	0.0724 (13)	0.0526 (11)	0.0454 (10)	−0.0008 (10)	0.0143 (9)	−0.0043 (9)
C1	0.0827 (16)	0.0550 (13)	0.0837 (17)	−0.0096 (11)	0.0138 (13)	0.0068 (12)
C4	0.0901 (16)	0.0577 (13)	0.0476 (11)	−0.0027 (11)	0.0130 (11)	−0.0034 (9)
C3	0.1043 (19)	0.0523 (12)	0.0586 (13)	−0.0098 (12)	0.0126 (12)	−0.0079 (10)
C7	0.0866 (16)	0.0671 (14)	0.0573 (13)	−0.0084 (12)	0.0348 (12)	−0.0037 (10)
C8	0.111 (2)	0.144 (3)	0.0552 (15)	−0.011 (2)	0.0182 (15)	0.0164 (17)

### Geometric parameters (Å, °)

**Table:**

O1—C11	1.249 (2)	C16—H16	0.93
O3—C17	1.364 (2)	C9—H9	0.93
O3—H3O	0.82	C13—H13	0.97 (2)
N1—C2	1.324 (3)	C15—H15	0.93
N1—C6	1.345 (2)	C6—C5	1.398 (3)
N3—C12	1.344 (3)	C18—C19	1.384 (3)
N3—N4	1.378 (2)	C18—H18	0.93
N3—H3N	0.91 (2)	C5—C4	1.395 (3)
N4—C13	1.275 (3)	C2—C3	1.394 (3)
O2—C12	1.229 (2)	C2—C1	1.502 (3)
N2—C9	1.340 (3)	C19—H19	0.93
N2—C6	1.382 (2)	C1—H1A	0.96
N2—C7	1.482 (2)	C1—H1C	0.96
C17—C18	1.380 (3)	C1—H1B	0.96
C17—C16	1.388 (3)	C4—C3	1.366 (3)
C14—C19	1.388 (3)	C4—H4	0.93
C14—C15	1.390 (3)	C3—H3	0.93
C14—C13	1.460 (3)	C7—C8	1.495 (4)
C11—C10	1.436 (3)	C7—H7A	0.97
C11—C5	1.462 (3)	C7—H7B	0.97
C10—C9	1.371 (3)	C8—H8B	0.96
C10—C12	1.483 (3)	C8—H8C	0.96
C16—C15	1.372 (3)	C8—H8A	0.96
			
C17—O3—H3O	109.5	C19—C18—H18	120
C2—N1—C6	117.86 (17)	C4—C5—C6	116.04 (18)
C12—N3—N4	120.71 (17)	C4—C5—C11	121.99 (18)
C12—N3—H3N	118.4 (15)	C6—C5—C11	121.97 (17)
N4—N3—H3N	120.9 (14)	N1—C2—C3	121.92 (19)
C13—N4—N3	115.59 (17)	N1—C2—C1	116.5 (2)
C9—N2—C6	119.42 (16)	C3—C2—C1	121.5 (2)
C9—N2—C7	120.42 (17)	O2—C12—N3	123.72 (19)
C6—N2—C7	120.13 (16)	O2—C12—C10	121.92 (18)
O3—C17—C18	118.75 (17)	N3—C12—C10	114.36 (17)
O3—C17—C16	121.44 (18)	C18—C19—C14	121.14 (19)
C18—C17—C16	119.80 (18)	C18—C19—H19	119.4
C19—C14—C15	117.65 (18)	C14—C19—H19	119.4
C19—C14—C13	121.51 (18)	C2—C1—H1A	109.5
C15—C14—C13	120.84 (18)	C2—C1—H1C	109.5
O1—C11—C10	124.68 (18)	H1A—C1—H1C	109.5
O1—C11—C5	120.61 (18)	C2—C1—H1B	109.5
C10—C11—C5	114.70 (16)	H1A—C1—H1B	109.5
C9—C10—C11	119.47 (18)	H1C—C1—H1B	109.5
C9—C10—C12	115.90 (17)	C3—C4—C5	119.9 (2)
C11—C10—C12	124.54 (17)	C3—C4—H4	120.1
C15—C16—C17	119.46 (18)	C5—C4—H4	120.1
C15—C16—H16	120.3	C4—C3—C2	119.8 (2)
C17—C16—H16	120.3	C4—C3—H3	120.1
N2—C9—C10	124.95 (18)	C2—C3—H3	120.1
N2—C9—H9	117.5	N2—C7—C8	112.4 (2)
C10—C9—H9	117.5	N2—C7—H7A	109.1
N4—C13—C14	120.13 (19)	C8—C7—H7A	109.1
N4—C13—H13	121.9 (13)	N2—C7—H7B	109.1
C14—C13—H13	117.9 (13)	C8—C7—H7B	109.1
C16—C15—C14	121.96 (18)	H7A—C7—H7B	107.9
C16—C15—H15	119	C7—C8—H8B	109.5
C14—C15—H15	119	C7—C8—H8C	109.5
N1—C6—N2	116.22 (17)	H8B—C8—H8C	109.5
N1—C6—C5	124.45 (18)	C7—C8—H8A	109.5
N2—C6—C5	119.32 (17)	H8B—C8—H8A	109.5
C17—C18—C19	119.94 (18)	H8C—C8—H8A	109.5
C17—C18—H18	120		
			
C12—N3—N4—C13	178.2 (2)	N2—C6—C5—C4	179.03 (18)
O1—C11—C10—C9	−175.46 (19)	N1—C6—C5—C11	−179.47 (18)
C5—C11—C10—C9	3.8 (3)	N2—C6—C5—C11	−0.3 (3)
O1—C11—C10—C12	1.1 (3)	O1—C11—C5—C4	−3.1 (3)
C5—C11—C10—C12	−179.61 (18)	C10—C11—C5—C4	177.57 (19)
O3—C17—C16—C15	−179.0 (2)	O1—C11—C5—C6	176.24 (19)
C18—C17—C16—C15	0.5 (3)	C10—C11—C5—C6	−3.1 (3)
C6—N2—C9—C10	−2.4 (3)	C6—N1—C2—C3	0.3 (3)
C7—N2—C9—C10	175.4 (2)	C6—N1—C2—C1	−178.45 (19)
C11—C10—C9—N2	−1.3 (3)	N4—N3—C12—O2	3.0 (3)
C12—C10—C9—N2	−178.10 (19)	N4—N3—C12—C10	−176.28 (17)
N3—N4—C13—C14	−179.25 (18)	C9—C10—C12—O2	−5.6 (3)
C19—C14—C13—N4	173.6 (2)	C11—C10—C12—O2	177.8 (2)
C15—C14—C13—N4	−5.2 (3)	C9—C10—C12—N3	173.73 (18)
C17—C16—C15—C14	1.4 (3)	C11—C10—C12—N3	−2.9 (3)
C19—C14—C15—C16	−2.4 (3)	C17—C18—C19—C14	0.4 (3)
C13—C14—C15—C16	176.5 (2)	C15—C14—C19—C18	1.5 (3)
C2—N1—C6—N2	−179.19 (17)	C13—C14—C19—C18	−177.4 (2)
C2—N1—C6—C5	0.0 (3)	C6—C5—C4—C3	−0.1 (3)
C9—N2—C6—N1	−177.63 (18)	C11—C5—C4—C3	179.3 (2)
C7—N2—C6—N1	4.5 (3)	C5—C4—C3—C2	0.4 (4)
C9—N2—C6—C5	3.2 (3)	N1—C2—C3—C4	−0.5 (4)
C7—N2—C6—C5	−174.73 (19)	C1—C2—C3—C4	178.2 (2)
O3—C17—C18—C19	178.2 (2)	C9—N2—C7—C8	98.6 (3)
C16—C17—C18—C19	−1.4 (3)	C6—N2—C7—C8	−83.5 (3)
N1—C6—C5—C4	−0.1 (3)		

### Hydrogen-bond geometry (Å, °)

**Table:**

*D*—H···*A*	*D*—H	H···*A*	*D*···*A*	*D*—H···*A*
N3—H3N···O1	0.91 (2)	1.91 (2)	2.660 (3)	139 (2)
O3—H3O···O2^i^	0.82	1.90	2.721 (3)	179

Symmetry codes: (i) −*x*, −*y*−1, −*z*+1.

## References

[bb1] Blessing, R. H. (1995). *Acta Cryst.* A**51**, 33–38. 10.1107/s0108767394005726 7702794

[bb2] Catalano, V. J., Kar, H. M. & Bennett, B. L. (2000). *Inorg. Chem* **39**, 121–127. 10.1021/ic9908827 11229018

[bb3] Chen, Y.-L., Fang, K.-C., Sheu, J.-Y., Hsu, S.-L. & Tzeng, C.-C. (2001). *J. Med. Chem* **44**, 2374–2378. 10.1021/jm0100335 11428933

[bb4] Farrugia, L. J. (1997). *J. Appl. Cryst.* **30**, 565.

[bb5] Farrugia, L. J. (1999). *J. Appl. Cryst.* **32**, 837–838.

[bb6] Ferrarini, P. L., Mori, C., Badawneh, M., Calderone, V., Greco, R., Manera, C., Martinelli, A., Nieri, P. & Saccomanni, G. (2000). *Eur. J. Chem* **35**, 815–819. 10.1016/s0223-5234(00)00173-2 11006483

[bb7] Gavrilova, A. L. & Bosnich, B. (2004). *Chem. Rev* **104**, 349–383. 10.1021/cr020604g 14871128

[bb8] Goswami, S. & Mukherjee, R. (1997). *Tetrahedron Lett* **38**, 1619–1622.

[bb9] Hoock, C., Reichert, J. & Schmidtke, M. (1999). *Molecules*, **4**, 264–271.

[bb10] Mintert, M. & Sheldrick, W. S. (1995). *J. Chem. Soc. Dalton Trans* pp. 2663–2669.

[bb11] Nakatani, K., Sando, S. & Saito, I. (2000). *J. Am. Chem. Soc* **122**, 2172–2177.

[bb12] Nakataniz, K., Sando, S., Kumasawa, H., Kikuchi, J. & Saito, I. (2001). *J. Am. Chem. Soc* **123**, 12650–12657. 10.1021/ja0109186 11741430

[bb13] Rigaku/MSC (2005). *CrystalClear* Rigaku/MSC, The Woodlands, Texas, USA.

[bb14] Roma, G., Braccio, M. D., Grossi, G., Mattioli, F. & Ghia, M. (2000). *Eur. J. Med. Chem* **35**, 1021–1026. 10.1016/s0223-5234(00)01175-2 11137230

[bb15] Sheldrick, G. M. (2008). *Acta Cryst.* A**64**, 112–122. 10.1107/S0108767307043930 18156677

[bb16] Zia-ur-Rehman, M., Anwar, J., Ahmad, S. & Siddiqui, H. L. (2006). *Chem. Pharm. Bull* **54**, 1175–1178. 10.1248/cpb.54.1175 16880664

[bb17] Zia-ur-Rehman, M., Choudary, J. A., Elsegood, M. R. J., Siddiqui, H. L. & Khan, K. M. (2009). *Eur. J. Med. Chem* **44**, 1311–1316. 10.1016/j.ejmech.2008.08.002 18804313

